# Recurrent Bilateral Optic Neuritis Associated with Myelin Oligodendrocyte Glycoprotein Antibody: A Case Report from Nepal

**DOI:** 10.1155/2021/8100423

**Published:** 2021-08-26

**Authors:** Sangam Shah, Rajeev Ojha, Sanjeeta Sitaula, Dosti Regmi, Ragesh Karn, Bikram Prasad Gajurel, Reema Rajbhandari, Niraj Gautam, Sunanda Paudel, Aashish Shrestha

**Affiliations:** ^1^Maharajgunj Medical Campus, Tribhuvan University Institute of Medicine, Maharajgunj, Kathmanadu 44600, Nepal; ^2^Department of Neurology, Tribhuvan University Institute of Medicine, Maharajgunj, Kathmanadu 44600, Nepal; ^3^Department of Ophthalmology, Tribhuvan University Institute of Medicine, Maharajgunj, Kathmanadu 44600, Nepal; ^4^Department of Radiology, Kanti Children's Hospital, Maharajgunj, Kathmanadu 44600, Nepal

## Abstract

Neuromyelitis optica spectrum disorder (NMOSD) is an immune-mediated inflammatory condition involving spinal cord and optic nerves. Diagnosis of NMOSD is done by aquaporin-4 antibody (AQP4) in patients with optic neuritis. Myelin oligodendrocyte glycoprotein (MOG) expressed on the oligodendrocyte cell surface and on the outermost cell surface of the myelin sheath may also be present in patients with NMOSD bilateral optic neuritis. Here, we describe a case of a thirty-nine-year-old-female with recurrent bilateral optic neuritis with positive anti-MOG antibody, and anti-MOG syndrome has not previously been reported from Nepal.

## 1. Introduction

Neuromyelitis optica spectrum disorder (NMOSD) associated with myelin oligodendrocyte glycoprotein (MOG) antibody is an immune-mediated inflammatory condition involving spinal cord and optic nerves, similar to NMOSD with aquaporin-antibody (AQP4) [1]. However, NMOSD with AQP4 antibody is expressed in astrocytes, and MOG antibodies are related to MOG of oligodendrocytes and myelin sheaths [2]. Here, we describe a case of a thirty-nine-year-old-female with recurrent bilateral optic neuritis with positive anti-MOG antibody and anti-MOG syndrome which have not previously been reported from Nepal.

## 2. Case Presentation

A 39-year-old female was admitted to our hospital with the chief complaint of blurring of vision of bilateral eyes for two weeks. It was nonpainful, without diplopia which was progressive for seven days, but the symptoms improved after that. The blurring of vision was noticed first in the right eye and then on the left eye in few hours of time. She had no headache, loss of consciousness, or limb weakness. She had similar two episodes of visual impairments in the past: first episode was four years back when she had a complete loss of vision, which was first noticed in the left eye and then in the right eye. Visual acuity at the time of presentation in a local hospital was 6/24 and 6/18 in the right and left eye, respectively. She also had headache with painful eyes on movement. The vision was fully recovered after fifteen days of medication then. The second episode was two years ago when she had sudden blurring of vision with a visual acuity of 6/18 and 6/12 in the right and left eye, respectively. There was no headache or painful eye-movements then. She was discharged in 5 days of admission with visual acuity of 6/9 in both eyes. Our patient is a regular smoker, 4–6 sticks for 10 years. There is no history of hypertension, diabetes, or peripheral vascular disease.

**Figure 1:**
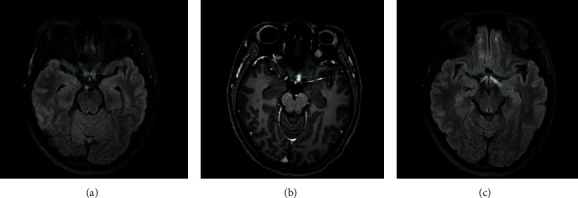
(a): FLAIR Axial image showing thickening and increased signal intensity in the optic chiasm. (b): T-1 weighted post Gadolinium contrast Axial image showing enhancement in the left side of the optic chiasm. (c): FLAIR Axial image showing increased signal intensity in the bilateral optic tracts. (Left > right).

On examination, visual acuity was 6/12 in the right eye and 6/9 in the left eye. Bilateral pupils were round, regular, and reactive (brisk, direct, and consensual reflexes were present). The rapid pupillary afferent defect was absent, while near reflex was present in both eyes. Bilateral pale disc was found on fundus examination. Her higher mental function and cranial nerves examination was normal. She did not have nystagmus, and her extraocular muscles were normal. Her muscle tone, power, and deep tendon reflexes were normal in all four limbs. Her gait, coordination, and stance were normal with no meningeal signs. Laboratory findings such as complete blood count, chest X-ray, random sugar, thyroid function, and renal and hepatic function tests were normal. Brain and orbit magnetic resonance imaging (MRI) showed cystic encephalomalacia (14*∗*9 mm) with surrounding gliosis in the posterior aspect of the right frontal lobe and T2 FLAIR high signal intensity in the left side of the optic chiasma and bilateral optic tracts with heterogeneous enhancement in the left side of optic chiasma, features suggesting demyelinating changes with active phase in the left side of the optic chiasma (Figures 1(a)–1(c)). Based on these findings, she was diagnosed with recurrent bilateral optic neuritis. She was treated with intravenous methyl prednisone for 3 days followed by oral prednisolone and azathioprine. She was discharged in 5 days of admission with improved bilateral visual acuity to 6/9. On follow-up after 2 weeks, the patient presented with reports of strongly positive for anti-MOG IgG antibodies done by cell-based immunoassay with immunofluorescence method, with negative antinuclear antibody and anti-AQP4 antibody. On 2-month follow-up, her visual acuity improved to 6/9 on right and 6/6 on left.

## 3. Discussion

A MOG protein is expressed on the oligodendrocyte cell surface and on the outermost cell surface of the myelin sheath and hence can undergo autoimmune demyelination [3]. About 75% of the NMOSD patients tested positive for AQP4 antibody, but MOG antibody is detected only in a minority of AQP4 antibody negative cases [4]. Anti-MOG antibody remains undetected in serum in early and remission stages, while anti-AQP4 antibody remains for several years once detected [5]. Our patient was detected positive for anti-MOG when she had recurrence for the third time. This signifies the importance of timing of anti-MOG antibodies.

Optic neuritis in NMOSD differs from optic neuritis in multiple sclerosis and idiopathic optic neuritis by severe visual loss, bilateral optic nerve involvement, relapsing course, and poor response to corticosteroids. Optic papillitis with ocular pain and retrobulbar type without ocular pain are more common in patients with anti-MOG antibody and AQP4 antibody, respectively, as compared to idiopathic optic neuritis [6]. Our patient had frequent relapse, bilateral involvement with severe vision improvement at onset, and painful eye movement in second attack. However, she was not compliant to medications after she was kept on regular low-dose steroids following the second attack.

Compared to anti-AQP4 antibody NMOSD, optic neuritis lesions in anti-MOG antibody NMOSD are relatively longer but less likely to reach optic chiasma and are confined in the anterior portion of the optic nerve in MRI [7]. Our patient's MRI showed hyperintensity in bilateral optic tracts and left optic chiasma with heterogeneous enhancement in contrast demyelinating changes with active phase in the left side of the optic chiasma.

NMO double-positive anti-AQP4 antibody and anti-MOG antibody were also been reported which had severe visual impairments and did not respond well to steroids and incomplete improvement in visual acuity [8]. Relapses of optic neuritis are more common in antibody-positive cases than in antibody-negative cases [9]. NMOSD associated with MOG antibodies presents with steroid responsive disease, bilateral concurrent optic neuritis, or transverse myelitis. Our patient also had 3 attacks responded well to steroids with near complete improvement in recent follow-up.

## 4. Conclusion

The diagnosis of NMOSD should not be limited to seronegative or positive AQP4 antibody in a patient presenting with optic neuritis and could still be anti-MOG optic neuritis. Anti-MOG optic neuritis can present with recurrent symptoms of visual impairment and have a good response to steroids.
